# Optimization of Non-Thermal Plasma Treatment in an *In Vivo* Model Organism

**DOI:** 10.1371/journal.pone.0160676

**Published:** 2016-08-09

**Authors:** Amanda Lee, Abraham Lin, Kajol Shah, Harpreet Singh, Vandana Miller, Shubha Gururaja Rao

**Affiliations:** 1 A.J. Drexel Plasma Institute, Drexel University College of Engineering, Camden, NJ, 08103, United States of America; 2 Department of Pharmacology and Physiology, Drexel University College of Medicine, Philadelphia, PA, 19102, United States of America; University Paul Sabatier, FRANCE

## Abstract

Non-thermal plasma is increasingly being recognized for a wide range of medical and biological applications. However, the effect of non-thermal plasma on physiological functions is not well characterized in *in vivo* model systems. Here we use a genetically amenable, widely used model system, *Drosophila melanogaster*, to develop an *in vivo* system, and investigate the role of non-thermal plasma in blood cell differentiation. Although the blood system in Drosophila is primitive, it is an efficient system with three types of hemocytes, functioning during different developmental stages and environmental stimuli. Blood cell differentiation in Drosophila plays an essential role in tissue modeling during embryogenesis, morphogenesis and also in innate immunity. In this study, we optimized distance and frequency for a direct non-thermal plasma application, and standardized doses to treat larvae and adult flies so that there is no effect on the viability, fertility or locomotion of the organism. We discovered that at optimal distance, time and frequency, application of plasma induced blood cell differentiation in the Drosophila larval lymph gland. We articulate that the augmented differentiation could be due to an increase in the levels of reactive oxygen species (ROS) upon non-thermal plasma application. Our studies open avenues to use Drosophila as a model system in plasma medicine to study various genetic disorders and biological processes where non-thermal plasma has a possible therapeutic application.

## Introduction

Non-thermal plasma systems have been emerging as useful tools for various clinical applications [1]. Plasma is known to catalyze biochemical activities when applied on tissue and is able to regulate cellular processes such as proliferation, differentiation, and apoptosis [1]. This, in part, is due to the reactive oxygen and nitrogen species (ROS and RNS) generated by application of non-thermal plasma [2]. Most of the plasma research has been *in vitro* or *ex vivo*, which has led to investigation of potential applications such as disinfection of surfaces [3], promotion of hemostasis [4], enhancement of tissue regeneration [5], acceleration of wound healing [6], and for anti-cancer therapy [7] [8]. However, there has not been an extensive characterization of non-thermal plasma in *in vivo* model organisms. In order to validate the biological effect of non-thermal plasma on *in vivo* systems, we used *Drosophila melanogaster* as a model for our study.

Drosophila provides a genetically amenable and well-established system that has been used over the past 100 years [9]. It has a short life span and is easy to maintain, making the studies rapid, efficient, and reproducible. It carries homologs for several disease-related genes, and conserved signaling mechanisms, and allows extensive genetic manipulations [10]. Time- and tissue-specific gene knockdowns or gene overexpression, clonal systems provide a better system for studying the effect of plasma on disease models especially oncogenic pathways [11,12]. Moreover, with the ease of fly husbandry and available genetic technology, it is possible to carry out large scale studies involving non-thermal plasma. There is a need to validate various effects of non-thermal plasma that have been observed in *in vitro* and *ex vivo* systems, and to establish the safety of non-thermal plasma as a feasible therapeutic option in an *in vivo* system. Hence, Drosophila will make an excellent candidate for investigating effects of non-thermal plasma application in an *in vivo* model.

In this study, we standardized application distance, treatment time, and an appropriate dose of floating electrode microsecond-pulsed dielectric barrier discharge (mspDBD) non-thermal plasma for treatment of Drosophila (both larvae and adults). Furthermore, we studied its effect on various parameters such as life span, survivability, fecundity, movement etc. We discovered that treatment at 50 Hz for 10 seconds was most efficacious in stimulating an effect without changing the life cycle or affecting the factors mentioned above. We also explored a physiological system in Drosophila modulated by ROS, i.e. hematopoietic system, and show that application of plasma can induce blood cell differentiation by increasing ROS levels in the glands.

## Materials and Methods

All flies used were either Canton-S or Hemolectin Gal4 GFP (obtained from Bloomington stock center).

### A. Non-thermal plasma treatment

**Fig 1:**
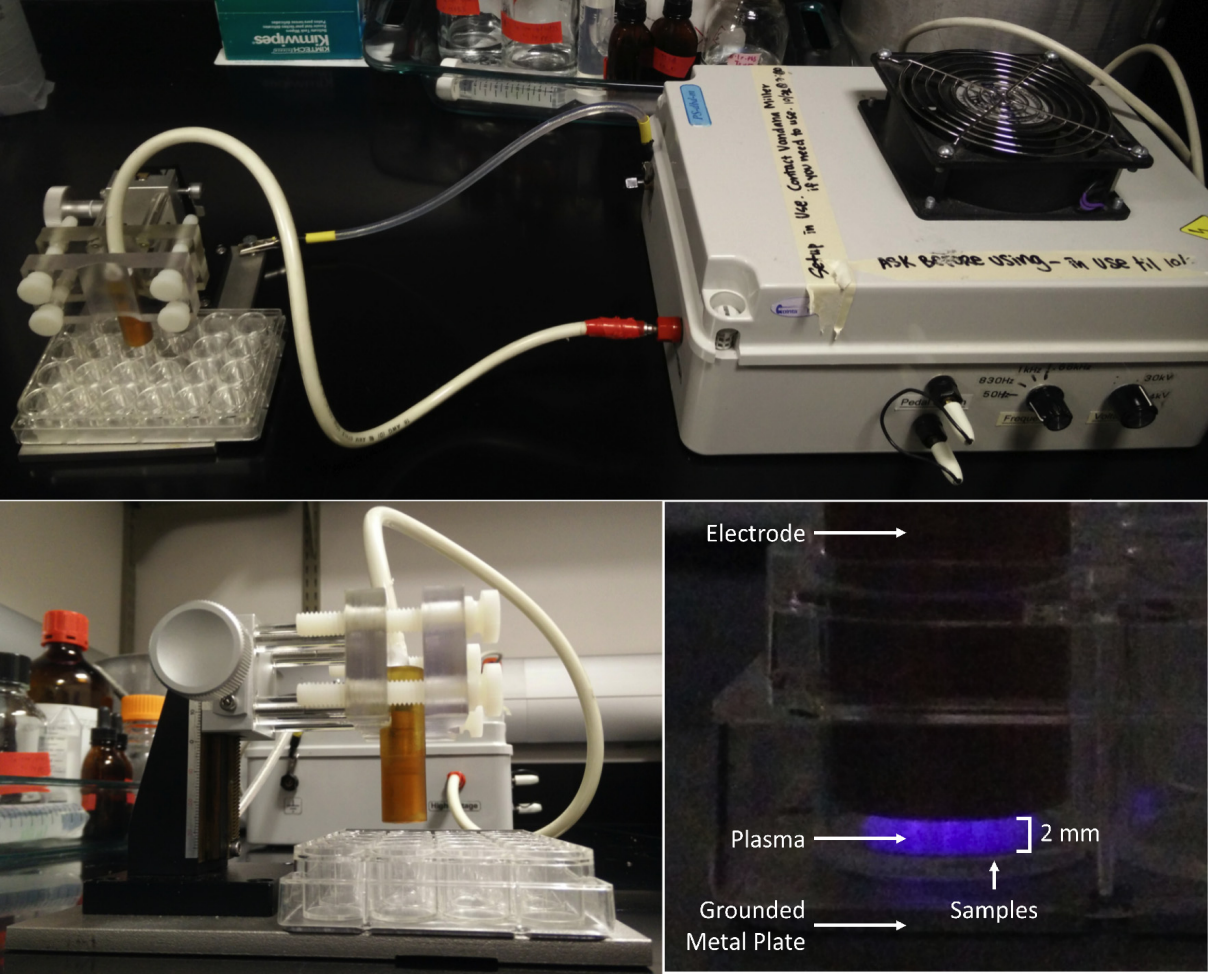
Experimental setup for plasma treatment of Drosophila (Top) High voltage electrode is connected to the mspDBD power supply and metal base of the z-positioner was grounded. (Bottom left) Z-positioner was used to position electrode 2 mm above the treated samples in the 24-well plate. (Bottom right) mspDBD discharge in a 24-well plate.

A mspDBD plasma was utilized for this study [13]. Plasma was generated by applying an alternating polarity pulse to a high-voltage, quartz-insulated electrode, 2 mm above the samples undergoing treatment “Fig 1”. Samples were placed into 24-well plates on top of a grounded metal base. The plasma pulse characteristics generated from the power supply are as follows: 30 kV amplitude pulse with rise times of 5 V/ns, pulse widths of 1.65 μs, and variable frequencies [13]. The energy per pulse of a single mspDBD discharge was established following the methods described in the previous published work [14]. Briefly, energy per pulse was calculated by measuring voltage and current, without displacement current, from a single discharge. This was found to be 0.5 mJ/pulse/cm^2^. The plasma treatment dose and total energy delivered to the cells during treatment, was calculated by multiplying the energy per pulse by the plasma treatment duration and frequency. All samples were treated for 10 seconds and the frequencies of pulses were 50, 850, and 1000 Hz, which corresponds to 0.012, 0.02, and 0.24 J per fly, respectively.

### B. Survivability

*D*. *melanogaster* were treated as adults (day 1) and larvae (early second instar) for multiple lengths of time as well as at various frequencies (50, 830 and 1000 Hz) to determine the optimal frequency and time period for treatment in the experiment. A total of 30 adults were treated for each time period of 5, 10, 15, 20, 25, 30, 35, 40, 45, 50 seconds and then the number of survivors were recorded after 3 days. Media was changed after every three days to record their life span.

Freshly hatched flies (males and females) were treated with plasma for 10 seconds and allowed to recover for 3 days in separate vials. One female and one male group (n = 5 groups) were mated in a vial and fecundity was tested at 3 different time points.

A negative geotaxis assay was performed, 2 days after treatment, by counting the number of flies that cross 5 cm mark in 18 seconds after tapping them to the bottom of the vial (5 flies each tested for five times).

Larvae were treated as second day instars. To ensure they were all at the same age, four plates were set up with plastic cages that allowed the flies to lay eggs for four hours. After 48 hours larvae were treated with plasma for the time periods of 5, 10, 15, 20, 25, 30, 45 and 50 seconds.

### C. Histochemistry

Two days after mspDBD plasma treatment, larvae were dissected and stained with DAPI (Sigma). Lymph glands were dissected in phosphate buffered saline (PBS) and then fixed for 20 minutes in 4% (*w/v*) paraformaldehyde (PFA). Samples were moved to PBS for 10 minutes and then washed in 0.1% (*v/v*) Triton-X 100 in PBS (PBS-T). Samples were incubated for 10 minutes in DAPI at 1:2000 dilutions in PBS. Tissues were washed in PBS-T for 10 minutes, and then mounted on slides with Mowiol (Sigma).

### D. ROS staining

Lymph glands were dissected quickly and placed in 6 μM dihydroethidium (DHE, Molecular probes) in PBS for 3 minutes and then washed in PBS three times for 3 minutes each. The samples were the fixed in 4% (*w/v*) PFA for 3 minutes and then washed again in PBS twice for 2 minutes each time. The lymph gland were then mounted in PBS and immediately photographed under a Zeiss confocal microscope. ROS levels were quantified by measuring integrated intensities [15] from treated and untreated lymph glands.

### E. ROS measurements

Larvae were homogenized using a pestle, and ROS was detected by amplex red using a fluorescence spectrophotometer (Hitachi F-2710) described previously [16,17]. Briefly, 5 μg horseradish peroxidase (Sigma-Aldrich) was added to the ROS buffer [mmoles/L, 20 Tris-HCl, 250 sucrose, 1 EGTA-Na_2_, 1 EDTA-Na_2_, pH 7.4 at 27°C] and the baseline fluorescence was measured (excitation at 560 nm and emission at 590 nm) for 30 minutes. Fluorescence was monitored continuously for 45 min at 5 s resolution and the rate of ROS production was calculated.

## Results

### A. Standardization of mspDBD plasma treatment in Drosophila

In order to standardize a dose to treat Drosophila with non-thermal plasma, we selected three frequencies (50 Hz, 830 Hz and 1 kHz) with varying time durations, using mspDBD “Fig 1”. We found that 1 kHz treatment immediately killed the flies. A 10 second treatment at 830 Hz made the flies partially immobile even after two days of recovery time. We observed that 50 Hz treatment did not affect the natural movement of flies, and hence decided to apply 50 Hz for increasing time durations to optimize time of exposure.

**Fig 2:**
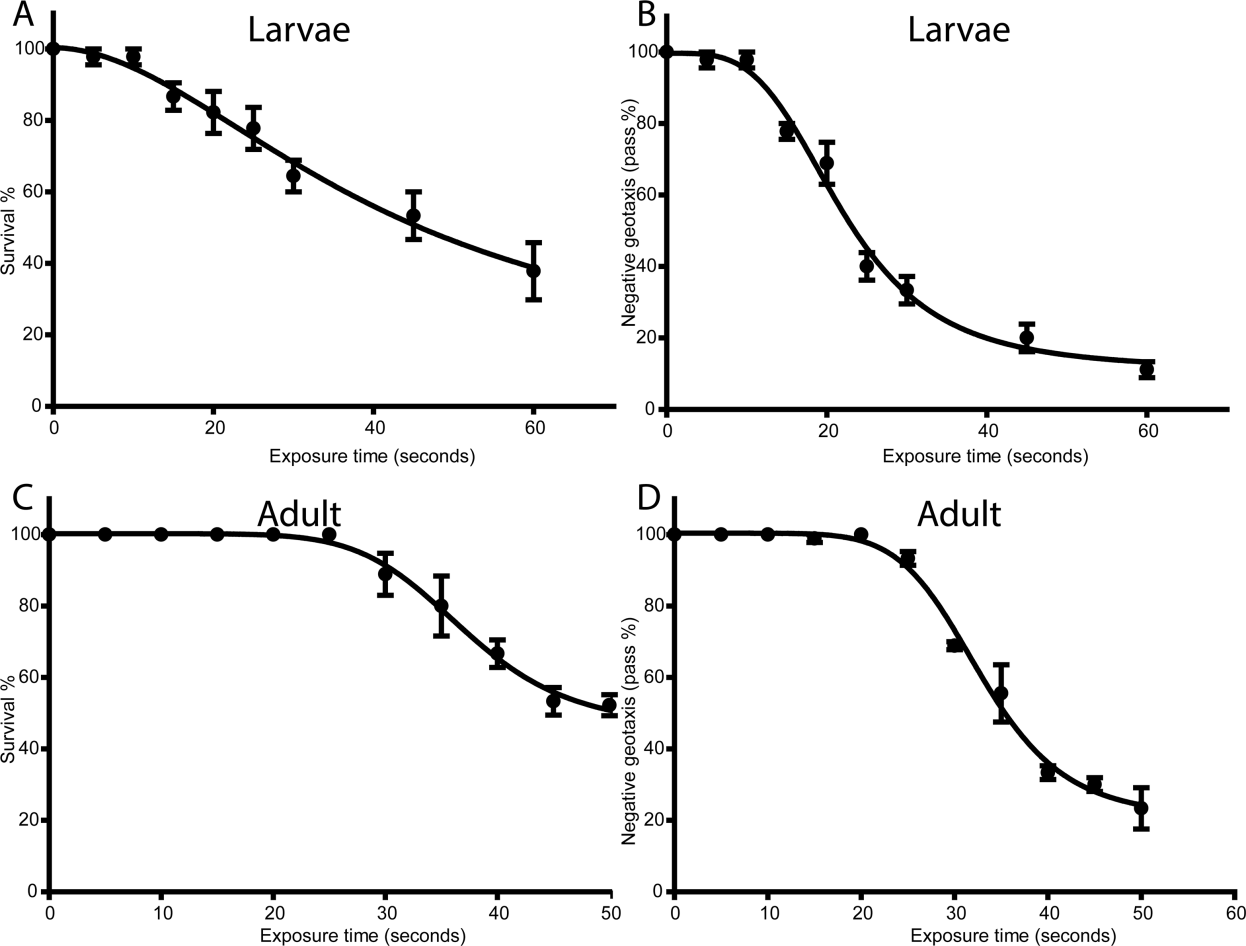
Standardization of non-thermal plasma doses in *Drosophila*. A and C: Graphs represent percentage of treated larvae or adults (A and C, respectively) survived at different time of 50 Hz treatment. B and D: Graphs represent negative geotaxis assay performed after 50Hz treatment of larvae after turning into adults and adults after 2 days after treatment.

We treated adult flies and larvae (30 each in five independent experiments) with mspDBD plasma at 50 Hz, for 0, 5, 10, 15, 20, 25, 30, 35, 40, 45 and 50 seconds, respectively. After exposure, adult flies and larvae were immediately transferred and cultured in fly-media. Larvae were let to eclose into adults to carry out tests for determining the effect of plasma. Treated adult flies were also maintained in the media for two days before carrying out further tests. We found that a treatment for 10 seconds was appropriate at which both treated larvae eclosed into adults and treated flies survived similar to their wild type counterparts “Fig 2A and 2C”.

### B. Physiology of mspDBD plasma treated Drosophila

After flies were recovered and cultured on fly media, they were tested for their physiological functions. Negative geotaxis was recorded for 15 flies (5 independent experiments) at room temperature to study their locomotory movements. “Fig 2B and 2D” shows negative geotaxis for flies (flies treated directly as well as treated larvae that eclosed into adults) after application of 50 Hz non-thermal plasma for different durations. The difference between treated and untreated groups of flies was calculated with student’s t-test. We found that application of non-thermal plasma for 10 seconds had no effect on negative geotaxis of flies.

Egg laying is a classical marker of physiological health of flies. In our fecundity test with flies treated for 10 seconds with non-thermal plasma, we found no significant difference in the number of eggs laid by controls and treated ones “S1 Fig”. Thus, we used 10 seconds as our standard treatment for all our subsequent experiments. Further, eggs from treated and untreated female flies hatched into adult flies.

Our results show that 50 Hz treatment for 10 seconds has no physiological impact on Drosophila and can be used for *in vivo* studies.

### C. mspDBD plasma induces blood cell differentiation

**Fig 3:**
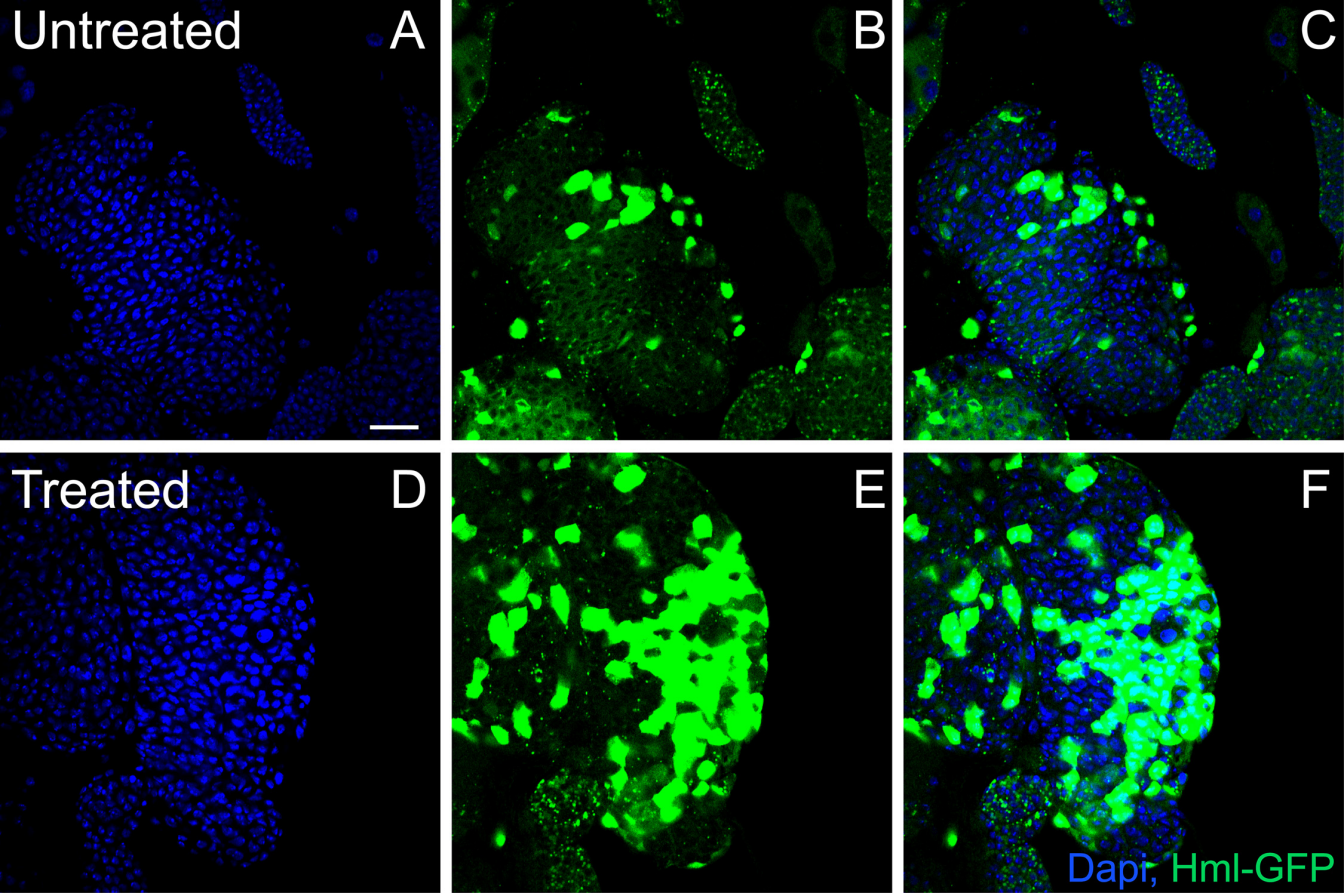
Plasma treatment increases blood cell differentiation. Treatment with plasma increased blood cell differentiation. Control (upper panel) and plasma treated larval lymph glands marked with DAPI (blue) for DNA and Hemolectin-GFP (green) to mark differentiated hemocytes. Scale bar is 25 μm.

Drosophila represents an extremely powerful system which can be easily modulated for identification of genes as well as signaling pathways crucial to the hematopoietic process that are also well-conserved in the vertebrate system. Hematopoiesis in Drosophila occurs in a specialized organ known as lymph gland [18]. The lymph gland is made up of three zones- a niche, a progenitor zone that exhibits increased levels of ROS, and a differentiated zone that has relatively lower levels of ROS [19]. Blood cell differentiation in flies is governed by several genetic pathways and is highly sensitive to changes in physiological factors. Lymph glands are extremely sensitive to changes in ROS levels [19] but it is not known if non-thermal plasma treatment increases ROS in lymph glands. Hence we tested whether application of mspDBD plasma will result in hematopoiesis in the fly lymph gland, given non-thermal plasma is known to generate reactive oxygen species (ROS) [20,21]. As predicted, application of mspDBD plasma resulted in increase in the number of differentiated cells (as measured by hemolectin GFP, a marker for differentiated hemocytes, after 48 hours of treatment) [22] “Fig 3” as compared to untreated flies.

### D. mspDBD plasma treatment increased ROS levels in lymph glands as well as whole larvae

**Fig 4:**
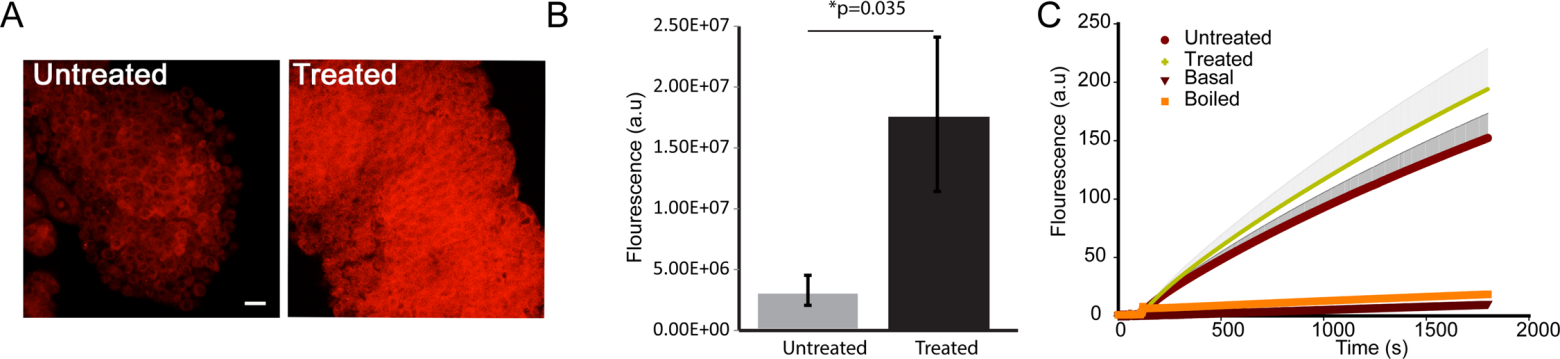
Plasma increased ROS levels in lymph glands and larvae. A: DHE staining of larval lymph glands (red) shows increase in ROS in control vs. plasma treated larvae. B: Quantification of ROS in larval lymph gland from A. C: ROS measurement of whole larval extracts using amplex red for treated, untreated, boiled and amplex red only samples. Values are normalized against protein concentration for each extract. Scale bar is 25 μm.

To directly address if mspDBD plasma treatment results in increase in ROS levels in lymph glands, we measured ROS in these tissues by staining with DHE, a superoxide indicator [19]. We observed that the lymph glands of Drosophila had significantly higher levels of ROS upon treatment with non-thermal plasma as compared to glands of untreated flies “Fig 4A and 4B” (p≤0.05). Diffused staining with DHE was observed in the entire lymph gland, indicating that ROS was generated throughout the gland. These results show that the increased blood cell differentiation is associated with increased levels in ROS, as it has been reported in catalase or superoxide dismutase mutants, which fail to scavenge the ROS in lymph glands [19]. Our results suggest that not only genetic manipulation of ROS *via* eliminating scavenging enzymes increases differentiation and ROS levels in the lymph gland [19], but also by external manipulation using mspDBD plasma.

We confirmed the increase in ROS levels by spectroflurometric measurements. To measure ROS, we prepared whole fly extracts and determined the amount of ROS in mspDBD plasma treated flies by using amplex red. As shown in the “Fig 4B”, control larvae and the boiled samples do not show detectable generation of ROS. However, larvae treated with plasma show increased ROS compared to the untreated wild type flies “Fig 4C”. These results indicate that there is a basal increase in ROS production upon treatment with mspDBD plasma.

Taken together, in this study, we establish a protocol and optimize conditions for the application of floating electrode mspDBD non-thermal plasma on Drosophila, at a level where their survival and reproduction is not affected. We tested the effect of plasma on hematopoietic organs and showed that there is an increased level of differentiation and this is due to augmented levels of ROS as a result of plasma treatment.

## Discussion

Dielectric barrier discharges (DBDs) that generate non-thermal plasma have been used for years for the generation of ozone and modification of surfaces among other applications [23]. DBDs applied directly to living cells and tissues have been demonstrated to kill bacteria, induce blood coagulation, and improved tissue regeneration [24–26]. Non-thermal plasma treatment has also been shown to affect cellular processes, such as augmenting endothelial cell proliferation and enhancing differentiation of stem cells [6] [27]. Along with the demonstrated clinical potential of plasma treatment, the simplicity and flexibility of DBD plasma systems make it appealing for clinical development [8]. However, in order to optimize this non-thermal plasma technology for clinical use, an understanding of the mechanisms underlying plasma effects is required. In this study, we characterized the physiological effects of an atmospheric pressure, floating electrode mspDBD plasma system in an *in vivo*, Drosophila model.

Drosophila is a powerful model organism used to study many disease models including cancer [28]. Here for the first time, we standardize the use of mspDBD plasma on Drosophila at a dose that does not compromise the viability or reproduction of the organism yet has an observable effect on its physiology. Drosophila also has well-characterized genetic and signaling pathways studied over the years especially ones that regulate growth, an aberrance in which leads to tumorigenesis [29]. Hence it would be a useful model system to test the effect of plasma in an intact organism for applications both in the areas of cancer biology and tumor immunology.

Our standardization methods identified a low dose of plasma regime that does not affect the viability, fecundity or the movement of animals. This dose is safe for both adults and larvae. Hence, it can be used to study effects of non-thermal plasma application on abnormal overgrowing tissues during larval growth phases or for treating already overgrown tissues in adults in the Drosophila oncogenic and other disease models. Our findings will also provide a baseline and a reference point for dosage and duration of mspDBD in other experimental models and organisms.

Drosophila lymph glands contain hemocytes that are blood cells of myeloid origin [30]. The mature hemocytes dissociate from the gland, flow in circulation and function as scavenging cells. We used this system to study the effect of non-thermal plasma on Drosophila because of its extreme-sensitivity to ROS levels. The progenitor cells of the lymph gland contain developmentally increased amounts of ROS and a further increase in plasma-stimulated ROS leads to increased differentiation as shown in the case of anti-oxidant mutant genetic backgrounds [19].

Our results conclude that mspDBD plasma treatment indeed increased the number of differentiated blood cells. We attribute this effect to an increase in ROS as we measured independently by spectrophotometric analysis and dye staining. These observations provide proof for our hypothesis that non-thermal plasma induces ROS generation that leads to increased differentiation possibly *via* JNK pathway as reported earlier [19]. The overall increase in ROS indicates a basal upregulation of ROS production. Hence, this could be an important therapeutic approach to cause death in tumor cells, given there are ROS mediated pathways contributing to apoptosis [31] [32]. Whether this ROS is generated by oxidases or mitochondria is to be explored in future studies which can further decipher the mechanism of ROS generation by mspDBD plasma.

When plasma is generated, four major components are produced that can be delivered to biological targets, including electric fields, UV radiation, charged and neutral gas species (e.g. peroxide, superoxide, and ozone) [33] [34] [35]. Recently, we have shown that active species from plasma trigger biological signaling to generate intracellular mitochondrial ROS [27] [36]. However, ozone produced in significant amounts (5–7%) from mspDBD [37] itself can trigger biological signaling pathways and induce ROS production [38]. Future work in this context will be to test individual components separately to determine the constituent causing the increasing in ROS in blood cells. However, based on previous work, we speculate that superoxide might be the major candidate for Drosophila blood cells given it is established that genetic depletion of superoxide dismutase enzyme increased ROS and differentiation [15].

In summary, our study has standardized the technique for atmospheric pressure, floating electrode mspDBD plasma treatment in an *in viv*o model organism, which can be used to study various physiological processes, disease models and phenotypes.

## Supporting Information

S1 Fig Fecundity of Drosophila after plasma treatment. Histogram represents the average number of eggs laid by untreated and plasma treated flies. There is no significant difference in the number of eggs laid.
(TIF)
