# Supplementary figures and images for: Optimization of Non-Thermal Plasma Treatment in an In Vivo Model Organism
Source: PLoS One. 2016 Aug 9;11(8):e0160676. doi: 10.1371/journal.pone.0160676 (PMC4978499; doi:10.1371/journal.pone.0160676)

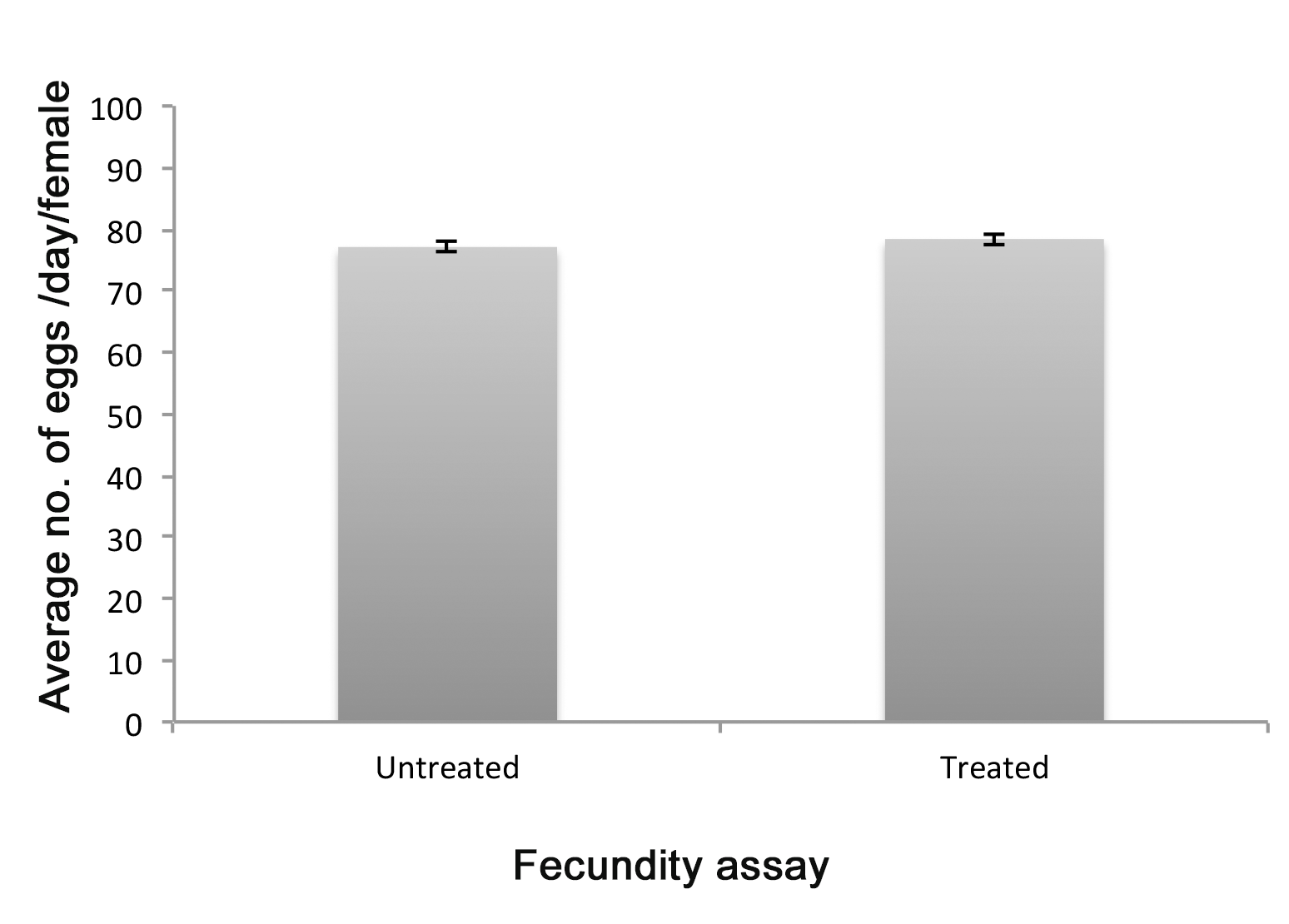

Supplement: S1 Fig — Histogram represents the average number of eggs laid by untreated and plasma treated flies. There is no significant difference in the number of eggs laid. (TIF) [file pone.0160676.s001.tif]
